# Irish potato imagery dataset for detection of early and late blight diseases

**DOI:** 10.1016/j.dib.2025.111549

**Published:** 2025-04-08

**Authors:** Hudson Laizer, Neema Mduma

**Affiliations:** The Nelson Mandela African Institution of Science and Technology, P O Box 447, Tengeru, Arusha, Tanzania

**Keywords:** *Solanum tuberosum*, Disease detection, Machine learning, Computer vision, Smallholder farming system, Image annotation

## Abstract

This dataset comprises of 58,709 annotated images of irish potato leaves, categorized into three classes (healthy, early blight and late blight). The data was collected over six months from smallholder farms in Southern Highlands Tanzania, using Samsung Galaxy A03 smartphones with 8-megapixel camera. Researchers, farmers and agricultural extension officers were trained to capture images under diverse conditions, including varying lighting, angles and backgrounds to ensure the dataset is diverse and representative. Plant pathologists were used to validate the images to ensure and enhance the reliability of the labels. Pre-processing steps such as duplicate removal, filtering of irrelevant images, annotation and metadata integration were applied resulting in a high-quality dataset. The dataset is organized into three folders (healthy, early blight and late blight) and is freely available on the Zenodo repository to promote accessibility for researchers working in the field of plant diseases. This dataset holds significant potential for reuse in training machine learning models for crop disease detection, transfer learning and data augmentation studies. By enabling early detection and classification of potato diseases, the dataset supports the development of innovative agricultural tools aimed at reducing crop losses and enhancing food security in Sub-Saharan Africa. Its robust design and regional specificity make it a valuable resource for advancing research and innovation in sustainable farming practices.

Specifications Table

**Table:** 

Subject	Computer Sciences
Specific subject area	Machine learning techniques for identifying diseases in Irish potatoes with a focus on early blight and late blight disease detection.
Type of data	Image
Data collection	Data were collected by researchers, farmers and agricultural extension officers using Samsung Galaxy A03 smartphones with an 8-megapixel camera equipped with the Open Data Kit (ODK) tool for capturing images. Images of potato leaves were taken under diverse conditions such as varying light, angles and backgrounds to ensure the dataset is representative. To control data quality, plant pathologists validated the images to ensure alignment with the corresponding disease classifications. Inclusion criteria focused on clear, unobstructed images of potato leaves, while irrelevant images were excluded during the data pre-processing. Additionally, the dataset primarily consists of images of the Shangi (Obama) potato variety, which is the most commonly cultivated in the study regions. Other varieties, such as Tengeru (Tigoni), were present but represented in smaller numbers. Images were taken at the most disease-susceptible stages in mid to late vegetative growth and early tuber bulking for early blight, and flowering to tuber initiation and bulking for late blight.
Data source location	Data were collected from farms located in Southern Highlands of Tanzania, specifically in Mbeya (8.9090° S, 33.4589° E), Iringa (7.7673° S, 35.6900° E), Njombe (9.3333° S, 34.7667° E) and Songwe (9.1333° S, 32.9333° E) regions.
Data accessibility	Repository name: ZENODO Data identification number: 10.5281/zenodo.8286529 Direct URL to data: https://zenodo.org/records/8286529

## 1 Value of the Data

• This dataset serves as a valuable resource as it addresses critical data gaps in the field of Artificial Intelligence in Agriculture by providing region-specific dataset with over 58,000 annotated images captured under diverse environment in the real-world smallholder farming conditions.
• This dataset can be reused in training machine learning algorithms for classification of early blight and late blight in Irish potato farming as well as transfer learning for other crops.
• By enabling early detection and classification of potato diseases, the dataset supports the development of innovative agricultural tools aimed at reducing crop losses and enhancing food security.
• The dataset is freely available in the Zenodo repository, promoting accessibility for researchers working in the field of plant diseases, which can accelerate research and innovation in sustainable farming practices.

## 2 Background

There is an urgent need to address the challenges faced by Irish potato smallholder farmers in Sub-Saharan Africa, particularly in Tanzania, where the crop serves as both food source and a significant cash crop [1,2]. Despite this importance, yields remain far below their potential, averaging to 8 t/Ha compared to optimal yields of 30–35 t/Ha [3]. This disparity is primarily due to diseases such as early blight and late blight, which can lead to yield losses of up to 50 % and 100 %, respectively [4,5]. Traditional methods of disease detection, which rely heavily on visual inspection by farmers and extension officers, are often prone to inaccuracies and delays, resulting in severe economic losses and food insecurity [6,7].

To address this issue, dataset was developed as part of broader effort to take advantage of machine learning and computer vision techniques for early and accurate detection of potato diseases. The theoretical foundation of this work is grounded in the transformative potential of data-driven approaches in agriculture, which enable timely interventions to mitigate crop losses [8,9]. However, most existing datasets are collected in controlled environments, which poses challenges for developing reliable machine learning models that require large, high-quality datasets from real-world environments. This dataset was collected under real-world smallholder farming conditions, incorporating diverse lighting, angles and backgrounds to enhance robustness. The dataset further provides a novel and valuable contribution to the field, especially for researchers and practitioners in resource-constrained agricultural settings, particularly in African contexts [10,11].

## 3 Data Description

**Fig. 1:**
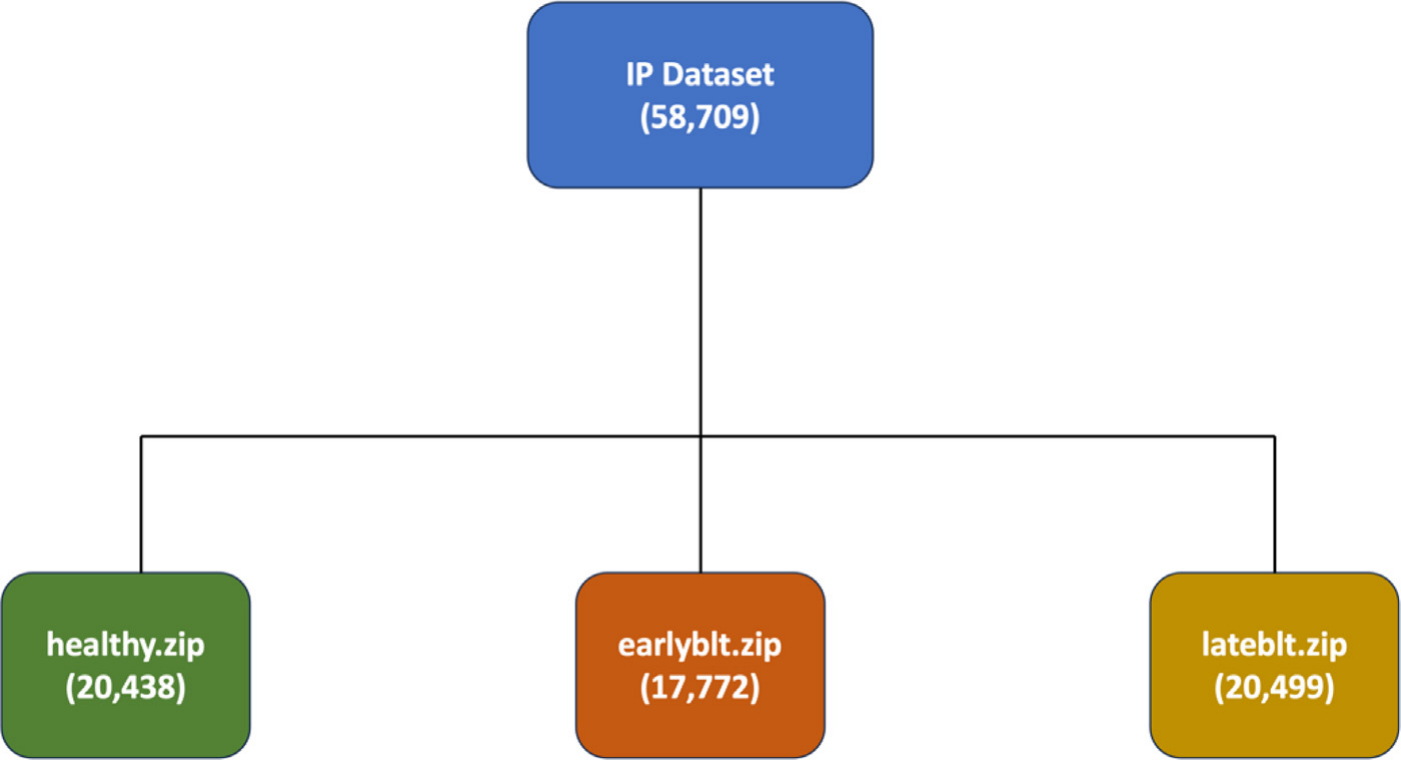
Dataset class folder information.

**Fig. 2:**
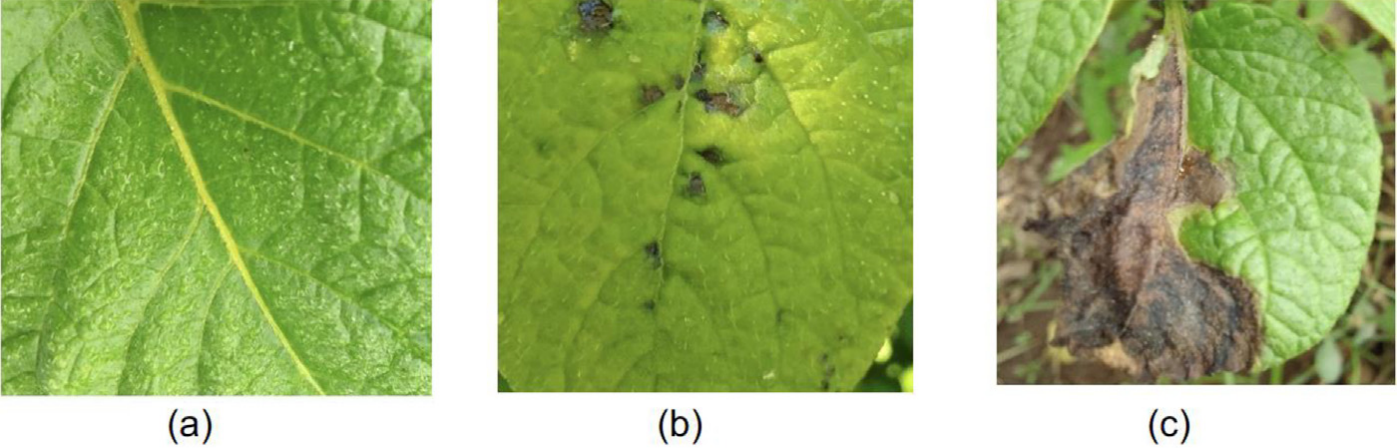
Images of Irish potato leaves (a) healthy (b) early blight (c) late blight classes.

The Irish potato (IP) dataset is organized into three primary zipped folders (Fig.1), namely healthy.zip, earlyblt.zip and lateblt.zip, each corresponding to a specific classification of potato leaf images (Fig.2) [12]. The healthy.zip folder contains 20,438 high-resolution images of potato leaves that are free from any visible disease symptoms. The earlyblt.zip folder comprises 17,772 images of potato leaves affected by early blight, a common fungal disease, characterized by distinctive brown spots. The lateblt.zip folder includes 20,499 images of leaves affected by late blight, a more severe fungal infection, often associated with large irregular lesions. Each image is stored in JPEG format and is systematically named to reflect its classification and a unique identifier for easy referencing and organization.

The dataset is further supplemented with a README file which outlines the structure and organization of the dataset, providing guidance for users on how to navigate and utilize the files effectively. For example, the README explains the criteria used for image classification, the naming conventions applied to each file and the metadata fields available.

## 4 Experimental Design, Materials and Methods

### 4.1 Data acquisition

The data collection process was overseen by the Mbeya University of Science and Technology (MUST) and the Tanzania Agricultural Research Institute (TARI). Researchers, farmers and agricultural extension officers were trained to use the Open Data Kit (ODK) application on Samsung Galaxy A03 Core smartphones equipped with an 8-megapixel camera to capture high-quality images. Plant pathologists played a crucial role in ensuring the accuracy of the images by validating them against healthy and diseased classes.

The Irish potato imagery was collected over six months, from 22nd November 2022 to 8th April 2023, in the Southern Highlands of Tanzania, specifically in Mbeya, Iringa, Njombe and Songwe regions. These regions were chosen due to the high prevalence of early blight and late blight diseases in Irish potato farming.

The dataset primarily features images from smallholder farms where the Shangi (Obama) variety is predominantly cultivated. Although other varieties like Tengeru (Tigoni) were present, they are less represented in the dataset. Images were captured during the most susceptible stages of Irish potato growth i.e. mid to late vegetative growth and early tuber bulking for early blight and flowering, tuber initiation and bulking stages for late blight.

### 4.2 Data preprocessing

The collected data from farms were preprocessed before uploaded to the Zenodo repository. Data preprocessing involved cleaning, labeling and annotating images of leaves which were temporarily stored in the Google drive. The data quality was double checked by plant pathologists to ensure validity of the image classes. Name of images were given by combining the class name and image number (i.e. “earlyblt12077.jpg” or “lateblt1566.jpg”). Data annotation was done by researchers with the help of algorithm developed by the project team which took into consideration the computer vision tasks specifically image classification. The mapping of the original filename and the new filename were stored in a dataframe along with other metadata about the image such as Global Positioning System (GPS) coordinates. Folders with class names were used to store images at their original dimensions. Table 1 presents number of Irish potato images before and after data preprocessing.

**Table 1:** Irish potato dataset before and after preprocessing.

Class name	Before preprocessing	After preprocessing
Healthy	20,947	20,438
Early blight	17,808	17,772
Late blight	21,016	20,499

## Limitations

The dataset presented in this article contains images of Irish potato leaves collected exclusively from farms in the Southern Highlands of Tanzania, specifically in Mbeya, Iringa, Njombe and Songwe regions. While the dataset is comprehensive in its focus on early and late blight diseases, it is limited to these two diseases and does not cover other potential potato diseases. Additionally, the images were captured under diverse but predominantly well-lit conditions to ensure high quality, thereby excluding images taken under poor lighting or with low-resolution devices. Moreover, the dataset primarily includes images of the most commonly cultivated potato variety in the study area, Shangi (Obama), with limited representation of other varieties, such as Tengeru (Tigoni).

The dataset also does not document the phenological stage of the potato at the time of image capture. Potato plants are susceptible to different fungal infections at various growth stages, with early blight primarily affecting plants from mid to late vegetative growth and early tuber bulking, while late blight is more severe during flowering, tuber initiation and bulking stages. While images were captured during the most vulnerable phenological stages, detailed records of plant developmental stages were not systematically included in the dataset. Future datasets could address these limitations by incorporating additional potato diseases, expanding to other regions of Tanzania, including a broader variety of potato cultivars and systematically documenting the phenological stage at the time of image capture. Additionally, capturing images under varied lighting conditions and with different imaging devices could improve dataset robustness and generalizability.

## Ethics Statement

Data for this study was collected from farms in the Southern Highland regions of Tanzania with informed consent obtained through a formal approval process. Farmers were actively involved in the study and provided consent by completing written forms, granting permission for data collection in their farms. The study adhered to ethical research standards, ensuring that participants agreed and were fully informed about the purpose and use of the collected data before the study commenced.

## CRediT author statement

**Hudson Laizer:** Conceptualization, Methodology, Software, Validation, Formal Analysis, Investigation, Resources, Data Curation, Writing - Original Draft, Writing - Review & Editing, Visualization, Supervision, Project Administration, Funding Acquisition. **Neema Mduma:** Conceptualization, Methodology, Writing - Review & Editing, Supervision, Project Administration.

## Data Availability

ZenodoIrish Potato Imagery Dataset for Early Detection of Crop Diseases (Original data).
